# Positive Information of COVID-19 and Anxiety: A Moderated Mediation Model of Risk Perception and Intolerance of Uncertainty

**DOI:** 10.3389/fpsyt.2021.715929

**Published:** 2021-08-03

**Authors:** Jun Zhao, Baojuan Ye, Tingting Ma

**Affiliations:** ^1^Center of Mental Health Education and Research, School of Psychology, Jiangxi Normal University, Nanchang, China; ^2^Mental Health Education and Counseling Center, Nanchang Hangkong University, Nanchang, China

**Keywords:** COVID-19, risk perception, intolerance of uncertainty, anxiety, moderated mediation model

## Abstract

Although COVID-19 information has been shown to play an important role in anxiety, little is known about the mediating and moderating mechanisms underlying this relationship. In the present study, we examined whether risk perception mediated the relationship between positive information of COVID-19 and anxiety and whether this mediating process was moderated by intolerance of uncertainty. A sample of 3,341 college students participated in this study and completed questionnaires regarding positive information of COVID-19, risk perception, intolerance of uncertainty, and anxiety. The results indicated that positive information of COVID-19 was significantly and negatively associated with anxiety and that risk perception partially mediated this relationship. Intolerance of uncertainty further moderated the relationship between positive information of COVID-19 and risk perception. Specifically, the relationship between positive information of COVID-19 and risk perception was significant for college students with low intolerance of uncertainty, while it became weaker for those with high intolerance of uncertainty.

## Highlights

- Positive information of COVID-19 was associated with anxiety.
- Risk perception mediated between positive information of COVID-19 and anxiety.
- Intolerance of uncertainty moderated the relationship between positive information of COVID-19 and risk perception.

## Introduction

Since 2020, the ongoing outbreak of coronavirus disease 2019 (COVID-2019) has swept the world. COVID-19 is an infectious disease caused by severe acute respiratory syndrome corona virus 2 (SARS-COV-2) with droplets and contact as the main modes of transmission (1). As a global case spike, the WHO has declared that COVID-19 is currently a global public health emergency (2). COVID-19 is characterized by high infectivity and high mortality. As of midnight on May 28, 2021, 169,172,262 cases had been confirmed, and 3,420,774 deaths resulted from COVID-19 globally. The widespread and rapid spread of COVID-19 has raised intense public attention and mental health stress, such as anxiety (3). Serious anxiety not only damages individuals' psychological function but also reduces individuals' immunity (4, 5). Immunity plays an important role in the prevention and treatment of COVID-19. Therefore, how to reduce the level of public anxiety during COVID-19 has become a question worthy of attention.

### Positive Information of COVID-19 and Anxiety

With the advent of the Internet 2.0 era, social media systems, dominated by Facebook, Twitter, and MicroBlog, have expanded rapidly, which makes it more convenient for people to access information. Since the COVID-19 outbreak, social media has become an important channel for people to know about COVID-19, and information about COVID-19 has been widely spread on social media. COVID-19, as a sudden risk event, will lead to the synchronous transmission of emotions with information as the carrier (6). The outbreak of Covid-19 has seriously threatened individuals' lives and life, so they need to obtain effective information. Wang (7) shows that the improvement of information effectiveness can relieve information anxiety. Shi et al. (8) found that positive SARS information, including recovery information with SARS and measures government took to prevent the spread of SARS, can improve individuals' mental health. Individuals will feel relieved after collecting and selecting valuable information from the massive epidemic information, which buffers the discomfort caused by traumatic events. Individuals having higher confidence in authority are associated with lower anxiety and individual having sufficient information about control measures beliefs in the ability to protect oneself and others are strongly associated with lower anxiety during COVID-19 epidemic (9). Trust in governmental actions to face COVID-19 and the subjective level of information regarding COVID- 19 are negatively associated with anxiety (10). The more positive information about the epidemic situation (such as the number of people cured, measures that are taken by the government) is obtained, the more certain they are about the safety of their environment, and they have more clear plans for their own lives, which may alleviate their anxiety. No study, to the best of our knowledge, has examined the relation between positive information of COVID-19 (such as the number of people cured, measures taken by the government) and anxiety among Chinese college students. Thus, the aim of the present study is to investigate whether positive information of COVID-19 (such as the number of people cured, measures taken by the government) is significantly associated with anxiety and examine the potential mediating and moderating mechanisms in this association.

### Risk Perception as a Mediator

Risk perception, defined as the subjective feeling and understanding of risk events, reflects the values and ideology of the individual (11, 12). When risk events (such as COVID-19) occur, the individual is affected by the information on risk events, and the individual will have subjective feelings and judgments on risk events and then produce a corresponding emotional experience and preparation behavior (13). Drawing from the Risk Information Seeking and Processing model (RISP) (14), we propose risk perception as a mediator between positive information of COVID-19 and anxiety. According to the RISP, after individuals acquire information on risk events, they will use the information to assess the severity of risk events and then generate corresponding emotional experiences. The model predicts that greater risk perception leads to increased negative emotion and decreased positive emotion (14). Anxiety is one of the most important emotional responses to risk perception (15). Although not yet tested, it is reasonable to expect that risk perception acts as a mediator between positive information of COVID-19 and anxiety. In the following section, previous research findings are reviewed to support this argument.

First, according to the cognitive model, information on risk events (such as COVID-19) will be associated with individuals' risk perception (16). A previous study showed that the more positive information an individual obtains about epidemic, the lower the risk perception (8). Second, high risk perception is more likely to develop high level of anxiety. The cognitive expectation theory of anxiety holds that anxiety is largely induced by the uncertainty of events and the severity of consequences (17). Individuals with a high level of risk perception generally believe that risk events are highly uncertain and uncontrollable and will bring serious consequences, which leads to anxiety (18).

### Intolerance of Uncertainly as a Moderator

Although positive information of COVID-19 may be significantly associated with anxiety through the mediating role of risk perception, not all individuals who are exposed to COVID-19 homogeneously experience a higher level of risk perception and show anxiety. Therefore, it is important to explore potential moderating variables that may influence the relationship between positive information of COVID-19 and anxiety.

The COVID-19 pandemic has created a high degree of uncertainty worldwide, and uncertainty distress is an understandable reaction. If uncertainty persist, it could become mental problem (19). Intolerance of uncertainty affects how individuals perceive, interpret, and respond to future uncertain situations (20) which is related to a variety of mental health problems (such as anxiety) (21) and plays a central role in the formation of generalized anxiety disorder (22, 23). Intolerance of uncertainty is a good indicator for clinical intervention (24) and is positively correlated with risk perception (25). The ecological theory proposes that individual development is the interaction between individuals and the environment, and individuals in the same environment will develop differently due to different individual characteristics (26). According to ecological theory, not all individuals who receive the same information about COVID-19 have the same level of risk perception, and the relationship between environmental factors (such as positive information of COVID-19) and development outcomes (such as risk perception) may be moderated by individual characteristics (such as intolerance of uncertainty). Tolerance of uncertainty refers to the set of negative and positive psychological response-cognitive, emotional, and behavioral-provoked by the conscious awareness of ignorance about particular aspects of the world, which is associate with health behavior and health outcomes (27). Intolerance of uncertainty (IU) refers to a dispositional negative orientation toward uncertainty and its consequences and is correlated with a tendency to react negatively on emotional, cognitive, and behavioral levels to uncertain and unpredictable situations (28). Intolerance of uncertainty is correlated with a failure to employ effective emotion regulation strategies, negative thoughts, and emotions about problems, and a perceived inability to cope effectively with aversive responses to uncertainty (29, 30).

Positive information of COVID-19 can help individuals more clearly understand COVID-19, which helps to reduce the uncertainty about COVID-19 and reduce risk perception. Positive information of COVID-19 is a protective factor for risk perception. The protective-limiting hypothesis (杯水薪) proposes that the environmental protective factor may lose its ability to counteract risk once the individual risk factor reaches a certain level (the protective effects of environmental factors are dampened in the face of high individual risk factors) (31). In this case, the beneficial effects of environmental factors will be stronger for individuals who have lower levels of risk factors. Hence, compared to college students with high intolerance of uncertainty, for college students with low intolerance of uncertainty, the relationship between positive information of COVID-19 and risk perception is stronger. Intolerance of uncertainty moderate the relationship between positive information of COVID-19 and risk perception such that high intolerance of uncertainty may weaken the association between positive information of COVID-19 and risk perception. To our knowledge, however, no previous studies have examined whether intolerance of uncertainty is a risk factor that moderates the relationship between positive information of COVID-19 and risk perception. Then, we examined whether the relationship between positive information of COVID-19 and risk perception would be moderated by intolerance of uncertainty.

### The Present Study

**Figure 1:**
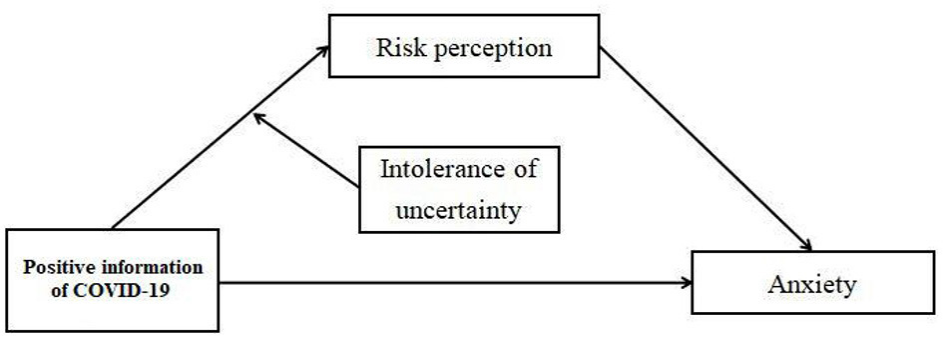
The proposed theoreticalmodel.

Taken together, the aims of the current study were 2-fold. First, the current study tested whether risk perception would mediate the relationship between positive information of COVID-19 and anxiety. Second, we tested whether intolerance of uncertainty would moderate the association between positive information of COVID-19 and risk perception (Figure 1). Based on the literature review, we proposed the following hypotheses:

**Hypothesis 1**. Positive information of COVID-19 would be positively correlated with anxiety.

**Hypotheses 2**. Risk perception would mediate the relationship between positive information of COVID-19 and anxiety.

**Hypotheses 3**. Intolerance of uncertainty would moderate the association between positive information of COVID-19 and risk perception.

## Methods and Materials

### Participants

After obtaining colleges and participants' consent, the research team distributed anonymous questionnaires to 3,341 college students (1,128 males and 2,213 females) in China: 1,765 (52.83%) freshmen, 1,138 (34.07%) sophomores, 306 (9.15%) juniors, and 132 (3.95%) seniors. The mean age of the participants was 19.57 years (*SD* = 1.38).

### Instruments

#### Positive Information of COVID-19 Questionnaire

Positive information of COVID-19 was assessed using positive information of COVID-19 questionnaire. This questionnaire was developed for individuals in the Chinese population. Based on the previous research (8), the questionnaire included two dimensions: (1) positive information of COVID-19 and (2) information on preventive measures of COVID-19. We compiled the COVID-19 questionnaire comprising 14 items (e.g., “I will get COVID-19 information through official channels”). Participants rated the items on a five-point scale (1 = strongly disagree to 5 = strongly agree), with higher scores representing that individual get more positive information about COVID-19. Confirmatory factor analysis (CFA) of this questionnaire suggested that the two-factors model fit the data well: CFI = 0.91, TLI = 0.90, RMSEA = 0.04, 90% CI = [0.03, 0.06], SRMR = 0.03. In the present study, Cronbach's alpha for this questionnaire was 0.96.

### Risk Perception Scale

Risk perception was assessed using the Risk Perception Scale. This scale was developed for individuals in the Chinese population. We compiled the Risk Perception Scale, comprised of 10 items. The scale included two dimensions: (1) familiarity (6 items; e.g., “I understand the cause of COVID-19”) and (2) controllability (4 items; e.g., “The government has taken appropriate measures to deal with COVID-19”). The response scale ranges from 1 (strongly disagree) to 5 (strongly agree). Responses to all items were averaged, with higher scores indicating that the individual believes that COVID-19 is more uncontrollable. Confirmatory factor analysis (CFA) of risk perception scale suggested that the two-factors model fit the data well: CFI = 0.90, TLI = 0.90, RMSEA = 0.07, 90% CI = [0.06, 0.09], SRMR = 0.06. In the present study, Cronbach's alpha for this scale was 0.72.

### Intolerance of Uncertainty Scale (IUS-12)

The Intolerance of Uncertainty Scale is a 12-item scale that was developed by Carleton et al. (32). We used Chinese translation of the scale (33). Individuals rated each item (e.g., “Unforeseen events upset me greatly”) on a five-point scale ranging from 1 (not at all characteristic of me) to 5 (entirely characteristic of me). Higher total scores indicated higher levels of intolerance of uncertainty. The Chinese version of the Intolerance of Uncertainty Scale has been demonstrated to be reliable and valid (34, 35). In the present study, Cronbach's alpha for this scale was 0.92.

### Self-Rating Anxiety Scale

Anxiety was measured using Chinese version of the Self-rating Anxiety Scale (36). The scale contains 20 self-reported items (e.g., “I feel more nervous and anxious than usual”). It is a 4-point scale ranging from 1 (none or a little of the time) to 4 (most or all of the time). Higher scores indicated higher levels of anxiety. The Chinese version of the Self-rating Anxiety Scale has been demonstrated to be reliable and valid (37). In the present study, Cronbach's alpha for this scale was 0.75.

### Procedure

This investigation was approved by the first author's University Ethics Committee. We obtained consent from all participants. Students were invited to participate in the survey anonymously and free to withdraw from the study at any time. Since this study was conducted during the COVID-19 pandemic, data collection was conducted *via* the Internet from February 9 to March 1, 2020. As an incentive for the participants, they received a small gift after the surveys were completed.

### Statistical Analysis

First, data screening revealed that there were no outliers in our data, and responses with missing data were excluded from data processing. Second, the study presented descriptive statistics and Pearson correlations for variables of interest. Mplus 7.4 was applied to examine the hypothesized moderated mediation model, and we evaluated the following goodness of fit indices to assess the adequacy of the model: the root mean square error of approximation (RMSEA), the comparative fit index (CFI), the Tucker-Lewis index (TLI), and the standardized root mean square residual (SRMR). Values >0.95 for the CFI and TLI, values <0.08 for the RMSEA, and SRMR are all considered an acceptable fit of the model to the data (38, 39). All study variables were standardized before structural equation modeling (SEM).

## Results

### Preliminary Analyses

Table 1 shows the means, standard deviations, and correlations of the variables. Positive information of COVID-19 was found to be negatively correlated with anxiety (*r* = −0.14, *p* < 0.001), which supported our first research hypothesis (Hypothesis 1). Risk perception was positively correlated with positive information of COVID-19 (*r* = −0.50, *p* < 0.001), intolerance of uncertainly (*r* = 0.16, *p* < 0.001), and anxiety (*r* = 0.28, *p* < 0.001). Intolerance of uncertainty was positively correlated with anxiety (*r* = 0.20, *p* < 0.01). Gender was correlated with anxiety. Age was correlated with positive information of COVID-19, risk perception, intolerance of uncertainty and anxiety. This result suggested that gender and age should be regarded as covariates in the next stage of analyses.

**Table 1:** Means, standard deviations, and correlations of the main study variables.

	**M**	**SD**	**1**	**2**	**3**	**4**	**5**	**6**
1. Age	19.57	1.38	1					
2. Gender	1.66	0.47	−0.08^**^	1				
3. Information	3.87	0.74	0.06^**^	−0.01	1			
4.Risk perception	2.23	0.45	−0.04^*^	−0.01	−0.50^***^	1		
5. Intolerance of uncertainty	2.75	0.70	0.09^***^	−0.02	−0.01	0.16^***^	1	
6. Anxiety	1.58	0.30	0.04^*^	−0.09^***^	−0.14^***^	0.28^***^	0.20^***^	1

*N = 3,341*.

* *P < 0.05*,

** *P< 0.01*,

*** *P< 0.001*.

### Testing for Mediation Effect and Moderated Mediation Effect

The random algorithm was used to parcel the items in each scale as indicators for each latent variable and each scale was packaged into 3 indicators in the present study. In the present study, age and gender were used as covariates in the structural equation model analysis. We used maximum likelihood to test the mediation model and moderated mediation model. The theoretical mediation model and moderated mediation model were examined with Mplus 7.4 (40). The results of the structural equation model analyzing the model showed that the mediation model (CFI = 0.95, TLI = 0.94, RMSEA = 0.06, 90% CI = [0.04, 0.07], SRMR = 0.05) and moderated mediation model (CFI = 0.95, TLI = 0.93, RMSEA = 0.07, 90% CI = [0.05, 0.08], SRMR = 0.06) fit the data well. The results showed that the values for the fit indices were excellent (38, 39).

According to this mediation model, positive information of COVID-19 was negatively related to college students' risk perception (γ = −0.69, *t* = −33.12, *p* < 0.001). Moreover, risk perception was positively related to college students' anxiety (γ = 0.12, *t* = 5.10, *p* < 0.001). This result indicated that risk perception mediated the relationship between positive information of COVID-19 and college students' anxiety. This model indicated that positive information of COVID-19 was directly related to college students' anxiety (γ = −0.07, *t* = −2.70, *p* < 0.01); therefore, risk perception partially mediated the relationship between positive information of COVID-19 and anxiety, which supported our second research hypothesis (Hypothesis 2). The effect size of the mediation effect was 0.53.

**Figure 2:**
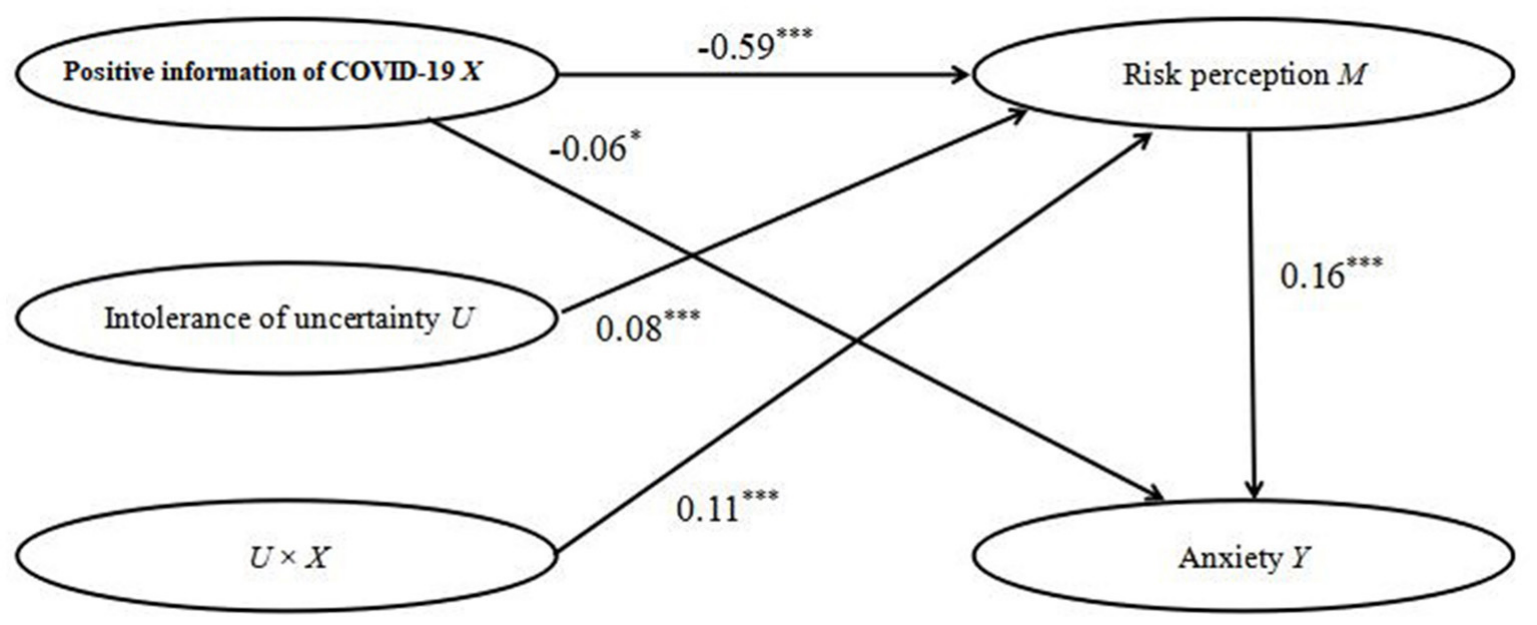
Moderated mediation result. *p < 0.05, ***p < 0.001.

According to this moderated mediation model presented in Figure 2, positive information of COVID-19 was negatively related to college students' risk perception (γ = −0.59, *t* = −34.11, *p* < 0.001). Moreover, risk perception was positively related to college students' anxiety (γ = 0.16, *t* = 5.40, *p* < 0.001). This result indicated that risk perception mediated the relationship between positive information of COVID-19 and college students' anxiety. Moreover, this model indicated that positive information of COVID-19 was directly related to college students' anxiety (γ = −0.06, *t* = −2.14, *p* < 0.05); therefore, risk perception partially mediated the relationship between positive information of COVID-19 and anxiety. Moreover, the interaction of positive information of COVID-19 and intolerance of uncertainty was significantly related to risk perception (γ = 0.11, *t* = 5.55, *p* < 0.001). Consistent with our third hypothesis (Hypothesis 3), intolerance of uncertainty moderated the relationship between positive information of COVID-19 and risk perception.

**Figure 3:**
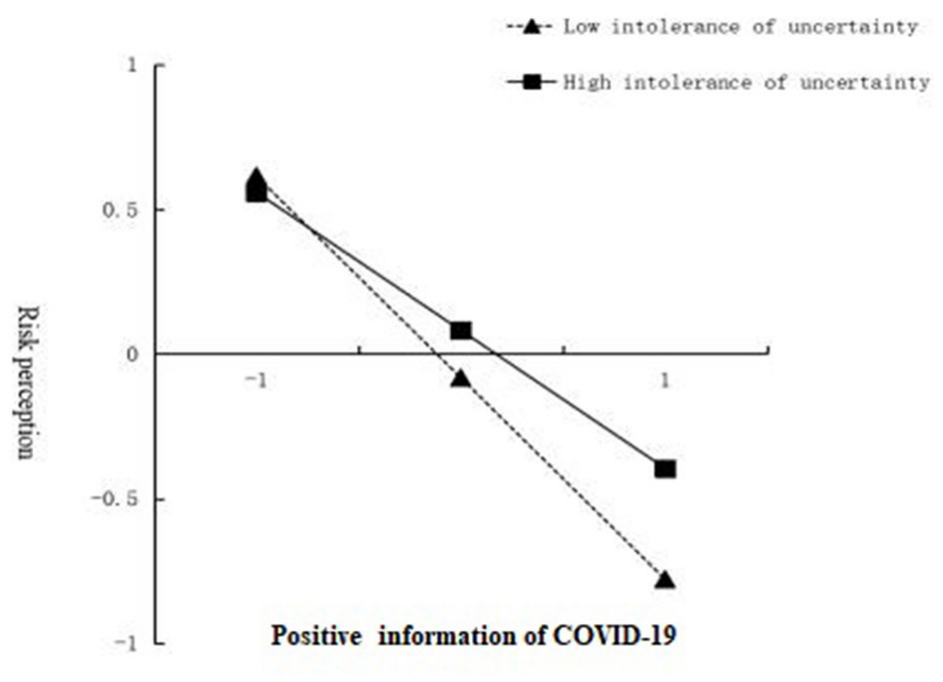
Association between positive information of COVID-19 and risk perception at higher and lower levels of intolerance of uncertainty.

To further portray the interaction, we conducted simple slope plots and calculated beta coefficients at −1 *SD* and +1 *SD* from the mean of risk perception. As depicted in Figure 3, for college students with a higher level of intolerance of uncertainty, the influence of positive information on risk perception was negative and statistically significant (*β* = −0.48, *t* = −22.14, *p* < 0.001). For college students with a lower level of intolerance of uncertainty, the influence of positive information on risk perception had a steeper slope, meaning it was more statistically significant (*β* = −0.70, *t* = −42.82, *p* < 0.001). This finding indicated that the negative relationship between positive information of COVID-19 and risk perception was greater for college students who had lower levels of intolerance of uncertainty compared to those with higher levels of intolerance of uncertainty, but these relationships remained negative overall.

## Discussion

The current study investigated the relationship between positive information of COVID-19 and individuals' anxiety. Our findings showed that positive information of COVID-19 was negatively related to anxiety. The access to more positive information of COVID-19 can increase individuals' confidence in overcoming the virus and can more positively look at the difficulties they are facing, and even make it possible for them to make a clear life and study plan (41, 42). One study suggests that more exposure to information was positively associated with anxiety throughout the EU during H5N1 (43). The reason may be as follows. First, the measurements of the epidemic information and anxiety were different. Van den Bulck and Custers (43) used TV viewing to measure exposure risk information. In their study, they used data from the European Audiovisual Observatory Yearbook 15 to obtain national estimates of average TV viewing per person per day, but anxiety was measured using self-report. Second, they assumed that those who watched a lot of TV also watch a lot of news, and the longer individuals watched TV, the more information they got about epidemic. As they said, TV viewing behavior showed that heavy TV viewers watched a lot of everything. As we all know, not all TV shows are about the epidemic such as TV play. In our study, we used positive information of COVID-19 questionnaire to measure positive information of COVID-19. The more information about the epidemic situation during the COVID-19 pandemic (such as the number of people cured, measures that are taken by the government, etc.) is obtained, the more certain individuals are about the safety of their environment, which can help people recognize the crisis and reduce their panic and anxiety.

Although the relationship between positive information of COVID-19 and anxiety is well-understood, the underlying mediation and moderation mechanisms are less clear. Our findings indicated that the beneficial effect of positive information of COVID-19 on anxiety was partially explained by risk perception. Furthermore, the relationship between positive information of COVID-19 and risk perception was moderated by the intolerance of uncertainty. The following sections discuss each of the research hypotheses in light of this mediation and moderation model of positive information of COVID-19 and anxiety.

### The Mediating Role of Risk Perception

The present study is the first to demonstrate the mediating role of risk perception in the association between positive information of COVID-19 and anxiety. That is, positive information of COVID-19 weakens risk perception about COVID-19, which in turn reduces anxiety among college students. Therefore, risk perception is not only an outcome of COVID-19 but also a protective factor against individuals' anxiety. Furthermore, it is worth noting that risk perception only partially mediated the relationship between positive information of COVID-19 and anxiety. The remaining direct and negative relationship between positive information of COVID-19 and anxiety may suggest that positive information of COVID-19 may function as a direct factor that can significantly reduce college students' anxiety.

In addition to the overall mediation result, each of the separate links in our mediation model is noteworthy. For the first stage of the mediation process (i.e., positive information of COVID-19 → risk perception), the present study found that positive information of COVID-19 was related to lower risk perception. This finding is consistent with the social amplification of the risk framework (44). That is, both the information source and nature of the information have an impact on risk perception. First, the more reliable the source of information, the more positive the individual perception of risk (45, 46). Second, positive information about an outbreak has a positive association with an individual's risk perception (8). The greater the impact of healing information and government preventive measures of COVID-19, the lower the level of individuals' risk perception. Throughout COVID-19, experts and government organizations have released authoritative and accurate information about COVID-19, which enables individuals to rationally assess risk events and generate positive risk perceptions.

For the second stage of the mediation model (i.e., risk perception → anxiety), the present study found that risk perception was associated with less anxiety. The reason may be as follows. First, individuals with low risk perception tend to believe that COVID-19 is controllable, which reduces the uncertainty of risk events and thus reduces individuals' anxiety (47). Second, the death reminder hypothesis holds that anxiety arises from the fear of death (48). During COVID-19, individuals' anxiety has primarily come from the risk of infection. When individuals have a positive risk perception of the epidemic, they tend to believe that the epidemic is controllable and preventive measures are effective, which reduces individuals' fear of infection with COVID-19 and thus reduces individuals' anxiety.

### The Moderating Role of Intolerance of Uncertainty

Our results also showed that intolerance of uncertainty moderated the relationship between positive information of COVID-19 and risk perception. This pattern is consistent with the protective-limiting model (31) and suggests that the effect of positive information of COVID-19 on risk perception is weaker for college students with a high rather than low intolerance of uncertainty. This result indicates that the protective effect of positive information of COVID-19 on individual development/risk perception is relatively sensitive, and the risk/negative effect of intolerance of uncertainty is relatively strong, which not only lead to an increase in risk perception but also to a weakening of the protective effect of epidemic information. There is a possible explanation for this finding. As individuals acquire more positive information about COVID-19, they perceive less uncertainty about COVID-19. However, not all individuals who receive the same positive information about COVID-19 have the same level of risk perception. For college students with a low intolerance of uncertainty, their tolerance of uncertainty for risk events is high. When they receive positive information of COVID-19, they tend to view the epidemic in a positive cognitive way and believe that COVID-19 is controllable, which leads to a stronger protective effect of epidemic information on risk perception. The reduction of uncertainty caused by epidemic information is more likely to reduce their risk perception. In contrast, college students with a high intolerance of uncertainty are more sensitive and negative to the uncertainty of COVID-19. When they receive positive information of COVID-19, they tend to view epidemic information in a negative cognitive way, which leads to the weakening of the protective effect of epidemic information. In other words, college students with a high intolerance of uncertainty benefit less from positive information of COVID-19 compared to those who have a low intolerance of uncertainty. To our knowledge, the present study is the first to confirm that intolerance of uncertainty as a moderator moderated the relationship between positive information of COVID-19 and risk perception. Therefore, our results filled this gap in understanding the relationship between epidemic information and risk perception.

### Limitations

There are also some limitations in the present investigation that need to be noted. First, the present study employed a cross-sectional design that does not allow for causal inferences. Future research should employ experimental and longitudinal designs to better explain the causal direction. Second, like any study based solely on self-report for data collection, there may have been response biases and social desirability effects. The results should be replicated with other, more comprehensive or even representative samples to achieve even more generalizable conclusions. Third, the present study was conducted in a sample of Chinese college students, which potentially limits the generalizability and indicates that similar research should be conducted in other types of samples.

Despite these limitations, the current study has several theoretical and practical contributions. From a theoretical perspective, this study further extended the previous research by confirming the mediating role of risk perception and the moderating role of intolerance of uncertainty. This would contribute to a better understanding of the relationship between positive information of COVID-19 and anxiety. From a practical perspective, with increasing positive information, college students maybe reasonably assess risk, and their anxiety maybe decrease. Government departments can conduct effective risk communication by releasing timely information about COVID-19, such as vaccine research progress and the infection rate, to help individuals establish risk perception rationally and reduce their anxiety. Moreover, college students with low level of intolerance of uncertainty may reduce their risk perception by disseminating more effective information. Compared to college students with low level of intolerance of uncertainty, for college students with high level of intolerance of uncertainty, they not only should get more positive information about the COVID-19 pandemic, but also reduce the level of intolerance of uncertainty in order to reduce risk perception.

## Data Availability Statement

The raw data supporting the conclusions of this article will be made available by the authors, without undue reservation.

## Author Contributions

JZ and TM wrote the manuscript. All authors contributed to the article, approved the submitted version, carried out the concepts, design, data acquisition, analysis, and manuscript editing.

## Conflict of Interest

The authors declare that the research was conducted in the absence of any commercial or financial relationships that could be construed as a potential conflict of interest.

## Publisher's Note

All claims expressed in this article are solely those of the authors and do not necessarily represent those of their affiliated organizations, or those of the publisher, the editors and the reviewers. Any product that may be evaluated in this article, or claim that may be made by its manufacturer, is not guaranteed or endorsed by the publisher.
